# Does LIN28B gene dysregulation make women more likely to abort?

**DOI:** 10.1530/RAF-21-0033

**Published:** 2021-08-12

**Authors:** QiaoYao Huang, YanRu Niu, LiJun Song, JinZhi Huang, Chenxi Wang, TianZhong Ma

**Affiliations:** 1 Reproductive Medicine Center, Affiliated Hospital of Guangdong Medical University, Zhanjiang, Guangdong, China; 2 Laboratory of Minimally Invasive Orthopaedics. Guangdong Medical University, Zhanjiang, Guangdong, China

**Keywords:** unexplained recurrent abortion, LIN28B, trophoblast, HTR-8/SVneo, BeWo, migration, invasion, apoptosis, fusion, miscarriage

## Abstract

**Background:**

LIN28B plays an important role in early embryonic development, but its role in villous trophoblast implantation and differentiation remains unknown. This study aims to verify the role of LIN28B in trophoblastic villous tissue and cells from women with URSA (unexplained recurrent spontaneous abortion) and artificial termination of pregnancy (negative control, NC).

**Methods:**

The *LIN28B* gene and its protein expression level were detected with real-time quantitative PCR, Western immunoblotting analysis, and immunocytochemistry. The gene was also overexpressed in chorionic villous cell lines (HTR-8/SVneo and BeWo) to examine its effect on trophoblast function.

**Results:**

The expression of LIN28B mRNA and protein of URSA villi was lower than that in the NC group. At the cellular level, overexpression of LIN28B enhanced cellular migration, and invasion, and inhibited apoptosis. LIN28B may inhibit apoptosis by promoting Akt phosphorylation and by inhibiting Bad phosphorylation and Bcl-2 expression. In addition, LIN28B inhibited cell fusion and reduced cellular syncytia.

**Conclusions:**

LIN28B can inhibit cell invasion and migration* in vitro* and promote apoptosis and fusion. The low expression of LIN28B in URSA villous trophoblast cells may be one of the causes of abortion. The role of LIN28B in villous trophoblasts needs further study.

**Lay summary:**

Propagation of offspring is of great significance to the continuation of the human race. However, continuous pregnancy is more difficult for some women, especially women who have multiple miscarriages. One important contributor is the cessation of development caused by genetic factors of the embryo, but there are still many unknown reasons. We investigated the LIN28B gene which is a possible pathogenic factor in the placenta. We collected 25 cases of abortion in the experimental group (unexplained recurrent abortion group) and 25 in the control group (artificial termination of pregnancy group): on average at 7–8 weeks of pregnancy. We tested the function of lin28b in these samples and verified its function in cell lines. LIN28B plays an important role in maintaining early pregnancy by promoting the invasion of villous cells, inhibiting apoptosis and fusion, and the reduction of LIN28B expression may lead to the occurrence of early miscarriage.

## Background

Recurrent spontaneous abortion (RSA 2012) refers to the failure of two or more pregnancies. Its etiology is complex and lacks specific clinical manifestations, with genetic factors comprising the main causes of early abortion (Ogasawara *et al.* 2000, Stephenson *et al.* 2002). However, with 50% of RSA still occurring due to unknown causes, we designate this as unexplained recurrent abortion (URSA) (RSA 2012). URSA is an excluded diagnostic disease, and its diagnosis and treatment still face great challenges, although studies have shown that the underlying causes of RSA include systemic endocrine and immune disorders (Mekinian *et al.* 2012, RSA 2012, Coomarasamy *et al.* 2015, Motedayyen *et al.* 2018, Canfield *et al.* 2019, Xu *et al.* 2019). Many scholars are now paying close attention to the gamete and the regulation of maternal–fetal interactions after blastocyst implantation (Bao *et al.* 2013, Knöfler & Pollheimer 2013, Xu *et al.* 2017). Gestation is a complex and delicate process and the maintenance of pregnancy depends upon the normal functioning of trophoblast cells. The fertilized oocyte subsequently develops into a blastocyst, with implantation usually occurring 6−7 days after fertilization. The trophectoderm (with a high differentiation potential) and the maternal endometrial epithelial cells undergo a series of complex interactions that allows embryonic contact, adherence, and implantation into the endometrium to ensure successful implantation. During this period, the primitive syncytium is generated by the fusion of early cytotrophoblastic cells. As the placental villi continue to develop, primary villi composed of cytotrophoblast and syncytiotrophoblast are formed (Zhou *et al.* 1997, Gamage *et al.* 2016). Normal functions, such as differentiation, migration, and invasion of chorionic trophoblast cells play an important role in the maintenance of pregnancy. A series of pregnancy-related diseases (including miscarriage and preeclampsia) may then be caused by the biologic dysfunction of trophoblast cells (insufficient differentiation, excessive apoptosis, impaired proliferation, or decreased invasiveness) (Huang *et al.* 2014, Windsperger *et al.* 2017, Wu *et al.* 2017, Yang *et al.* 2018, Canfield *et al.* 2019).

LIN28 was first found to be encoded by the *Lin28b* gene on chromosome 6q21 when the sequence gene was screened within *C. elegans* (Ambros and Horvitz 1984), and its expression is histologically specific, localized primarily in the placenta, testis, and fetal liver. There are two congeners of LIN28A and LIN28B in mammals: these are highly conserved RNA-binding proteins, and LIN28B is a highly conserved structural protein in advanced eukaryotes (Thornton & Gregory 2012, Shyh-Chang & Daley 2013). Although LIN28B RNA-binding proteins play an important role in embryonic development and implantation (Lozoya *et al.* 2014, Zhao *et al.* 2018), the mechanism of action in the chorionic trophoblast of URSA remains unclear. We herein explore a possible role for LIN28B in placental trophoblast in URSA patients at the tissue and cellular levels.

## Methods

### Experimental object and specimen collection

#### Experimental object

The protocol governing the subjects of this study was approved by the Ethics Committee of the Affiliated Hospital of Guangdong Medical University (PJ2013014). Patients with unexplained recurrent abortion (URSA group) or unexpected pregnancy which refers to accidental pregnancy caused by the failure of contraceptive measures (usually refers to a married woman who has an unplanned pregnancy) (<12 weeks) (NC group) were selected from the Outpatient Department of the Affiliated Hospital of Guangdong Medical University from 2016 to 2018. Each group entailed 25 cases of chorionic villi. The mean age of the URSA group was 33.73 ± 5.33 years (mean ± s.d.), and the mean gestational age was 7.82 ± 0.81 weeks. The mean age of the NC group was 31.23 ± 4.83, and the mean gestational age was 7.31 ± 0.95 weeks. All of the patients signed informed consent forms. The inclusion criteria for the URSA group were all of the patients who underwent two or more miscarriages, possessed excluded possible causes (chromosomal abnormalities, immunological factors, etc.), and were diagnosed as having early abortion villi of URSA (<12 weeks). For the NC group, chronologic and gestational age were similar, and the patients who voluntarily requested termination of pregnancy had a history of normal pregnancy and delivery before the current pregnancy, had no stillbirths, no history of spontaneous abortion, no medication use, and no history of viral infection. During the current pregnancy, there were no threatened abortion symptoms or signs, and B-ultrasonography suggested normal embryonic development. There was no statistical difference in age and gestational age between the two groups (*P*  > 0.05).

#### Specimen collection

The villi were extracted immediately in the aseptic state, washed with sterile saline until no obvious blood was observed. The villi were divided into three parts (some of the villi were quickly placed into cryopreserved tubes containing 1 mL of Trizol reagent (Invitrogen)). Tissues were placed into a cryotube, quickly deposited into liquid nitrogen for quick freezing, and stored in a –80°C refrigerator (Invitrogen) for later extraction of tissue protein and RNA. Partial fixation in 4% (v/v) formaldehyde was used for immunohistochemistry.

### Cell culture

HTR-8/SVneo or BeWo cells from the Stem Cell R&D and Clinical Transformation Center of the Affiliated Hospital of Guangdong Medical University were cultured in RPMI 1640 medium (Gibco) containing 10% (v/v) fetal bovine serum (FBS, Gibco) and placed in an incubator with a CO_2_ concentration of 5%, a humidity of 95%, and a temperature of 37°C. Different cell lines were developed to study trophoblast functions (cell fusion, migration, and invasion) including BeWo and HTR-8/SVneo. Studies have shown that BeWo is positive for the trophoblast/epithelial marker CK7, while HTR-8/SVneo cells contain almost no CK7 positive cell clusters. Studies have shown that BeWo is more used to study cell fusion, while HTR-8/SVneo is inclined to invasion and migration functions.

### Transfection

For HTR-8/SVneo or BeWo cells, Lipofectamine 3000 (Invitrogen) was used to transfect plasmids (a LIN28B overexpression plasmid was constructed with green fluorescent GFP, EX-Y3355-Lv105,GeneCopoeia, US). Transfection was carried out according to the specifications of the transfection reagent. Transfection efficiency was about 70%.

### Immunohistochemistry

Immunohistochemical staining using a primary antibody (LIN28B, Abcam) was performed on sections of placental villi. According to the instructions for the SP Rabbit and Mouse HRP Kit (DAB) (CWBIO, China), biotinylated anti-rabbit/mouse universal second antibody was combined with the first specific antibody, and the second antibody was labeled with biotin combined with the streptavidin-labeled peroxidase (HRP). A streptavidin complex was thereby achieved by labeling with antigen-specific first antibody-biotinylated second antibody-HRP. Blank control: the primary antibody was replaced with PBS, other steps were as previously mentioned (including endogenous peroxidase blocking and serum blocking). We used ImageJ software to carry out a sample image for immunohistochemistry and made quantitative comparisons using digital image analysis.

### RNA and protein extraction

We used collected tissue or untreated cell samples to which Trizol (Invitrogen) was added and ground and homogenized to extract RNA, while the protein was extracted by homogeneous lysis of RIPA lysate (Beyotime, China) containing 1% PMSF (Beyotime, China).

### Real-time quantitative PCR (qPCR)

cDNA was synthesized according to the manufacturer’s instructions found in the PrimeScriptRT Reagent Kit using gDNA Eraser (TaKaRa, Japan) kit, and the TB Green chimeric fluorescence method (TaKaRa, Japan) was used for real-time quantitative PCR. An ABI7500 real-time qPCR system was used to perform all qPCR reactions, and the data were standardized to β-actin. The qPCR primer sequence is shown in Table 1. We analyzed relative gene expression using the semi-quantitative 2^−ΔΔCt^ method.

**Table 1:** Primers used for qPCR experiments.

Gene	NCBI accession no.	Primer sequence (5′–3′)	
		Forward	Reverse
*LIN28B*	NM_001004317	CCAGCCATGCACTTCAACTCTCC	TGACCTGCCTGACCGTTCTGAG
*ACTB*	NM_001101	GGCACCACACCTTCTACAATGAGC	GATAGCACAGCCTGGATAGCAACG
*CDH1*	NM_004360	GCTCTTCCAGGAACCTCTGTGATG	AAGCGATGGCGGCATTGTAGG
*CDH3*	NM_001793	GAGAACCTGAAGGCGGCTAACAC	CTTGGTCGGAGGCGGAGGAG
*VIM*	NM_003380	TTGCCGTTGAAGCTGCTAACTACC	AATCCTGCTCTCCTCGCCTTCC
*CDH2*	NM_001792	AGGCGTCTGTAGAGGCTTCTGG	GAGGCTGTCCTTCATGCACATCC
*ERVW-1*	NM_001130925.1	CCCCATCGTATAGGAGTCTTTC	GTTTGGGTGAAGTAAGTCCAAC

### Western blotting analysis

As mentioned previously, total protein lysates were separated by PAGE, transferred to PVDF membranes (Millipore), and sealed at room temperature with 5% (w/v) milk (BD, US) for 2 h. Lysates were incubated with LIN28B (Abcam), β-actin, Akt, p-Akt, Bad, p-Bad, or Bcl-2 primary antibodies overnight at 4°C and then incubated with secondary antibody (CST,US) for 1 h at room temperature. The resulting immunoblot was scanned and quantitatively assessed using a Tanon 5200 imaging system.

### Invasion and migration assays

HTR-8/SVneo or BeWo cells were seeded in a 6-well culture dish and transfected with a plasmid on the next day. After 24 h, the transfected cells were digested and resuspended in serum-free RPMI 1640 medium and inoculated to an upper chamber (Corning) that had been coated with matrigel (Corning); the lower chamber was placed in 10% (v/v) FBS. After 1 or 2 days, the non-invasive cells in the upper chamber were wiped with a swab, and the remaining invasive cells were then stained with crystal violet and imaged. We counted five fields of view in each cell, and the numbers of invasive cells were analyzed statistically. The steps with respect to migration were the same as for invasion, but the inoculated upper chamber did not need to be covered with matrigel.

### Apoptosis assay

FACSCantoII flow cytometry (BD, US) was used to measure apoptosis according to the instructions in the PE Annexin V apoptosis kit maker (BD, US). Cells that are considered viable are PE Annexin V and 7-AAD negative; cells that are in early apoptosis are PE Annexin V positive and 7-AAD negative; cells that are in late apoptosis or already dead are both PE Annexin V and 7-AAD positive.

### Evaluation of cell cycle kinetics

According to the manufacturer’s instructions found in the cell cycle and apoptosis detection kit (Beyotime, China), we fixed cells with 70% (v/v) ethanol for 4 h at 4°C and then added pyridine iodide staining solution (for a 0.5 mL sample we used 25 μL of 20× staining buffer and 10 μL of 50× RNase A at 37°C for 30 min) and measured cell cycle parameters by FACSCantoII flow cytometry (BD, US) within 24 h.

### Cell fusion assay

BeWo cells were inoculated in confocal dishes and transfected with plasmid when the cell density was 30−50%. After 12 h, 50 μM adenylate cyclase activator (forskolin, FSK, Beyotime, China) and 0.1% (v/v) DMSO were added consecutively. After continuous culture for 1 or 2 weeks, we immobilized the cells with 4% (v/v) paraformaldehyde, incubated them overnight with β-catenin (Abcam) at 4°C, and then incubated them additionally with secondary antibody (CST) for 1 h at room temperature. After staining cells with DAPI, we used a laser confocal microscope (Olympus FV3000) to photograph and analyze results. The full field of view of the confocal dish was taken using a 20× objective, and the number of cells with ≥3 nuclear fusions was counted.

### Statistical analysis

The results are expressed as mean ± s.e. The normality and variance equivalence of all data were tested to determine the appropriate statistical test. We used Student’s *t-*test, *x*^2^, or Fisher's exact probability test to determine significant differences between groups. All statistical analyses were processed using GraphPad Prism 7 software and a difference of *P* < 0.05 was considered statistically significant, except where noted. The number of experimental replicates and the number of replicates within each experiment were both three times.

## Results

### The URSA group showed decreased expression of LIN28B mRNA and protein

**Figure 1:**
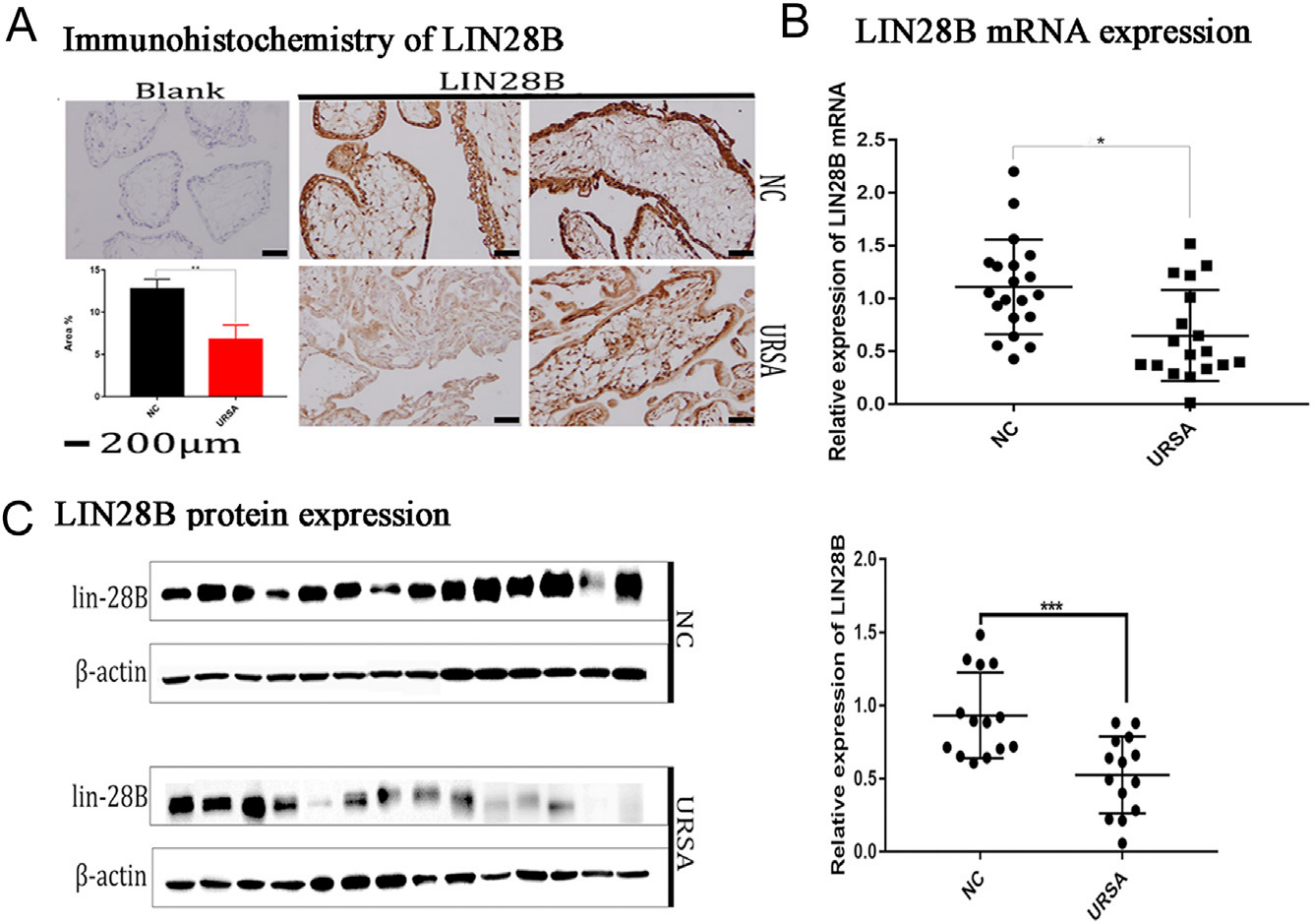
Low expression of LIN28B in placental villi of unexplained recurrent abortion (URSA). (A) Immunohistochemical analysis of LIN28B in NC villus trophoblast (middle and upper right) compared with URSA tissue samples (middle and lower right) and blank control (left), quantitative analysis of two groups (bottom left) (*n* = 25). Blank control: the primary antibody was replaced with PBS. (B) The relative expression of LIN28B mRNA in placental villi of URSA and NC (*n* = 20). (C) The expression of LIN28B protein in URSA and NC villi (*n* = 14). The number of experimental replicates and the number of replicates within each experiment were both three times. ****P < 0.0001, ***P < 0.001, **P < 0.01, *P < 0.05.

We verified the expression of LIN28B in URSA villi by IHC, WB, and qPCR. The results of IHC showed that the expression of LIN28B in the chorionic villi of the URSA group (6.76 ± 0.87) was lower than that of the NC group (12.75 ± 0.57) (*P* = 0.0012) and that LIN28B was expressed in the nucleus and cytoplasm of the villous epithelial tissue but not in the villous interstitial tissue (Fig. 1A). qPCR results showed that the LIN28B mRNA expression level was lower in the URSA group (0.53 ± 0.16) relative to the NC group (1.18 ± 0.21) (*P* = 0.003) (Fig. 1B). The WB results also showed that LIN28B protein in the URSA group (0.53 ± 0.07) was lower than that in the NC group (0.93 ± 0.07) (*P* = 0.007) (Fig. 1C).

### LIN28B can be overexpressed in HTR-8/SVneo and BeWo cells

**Figure 2:**
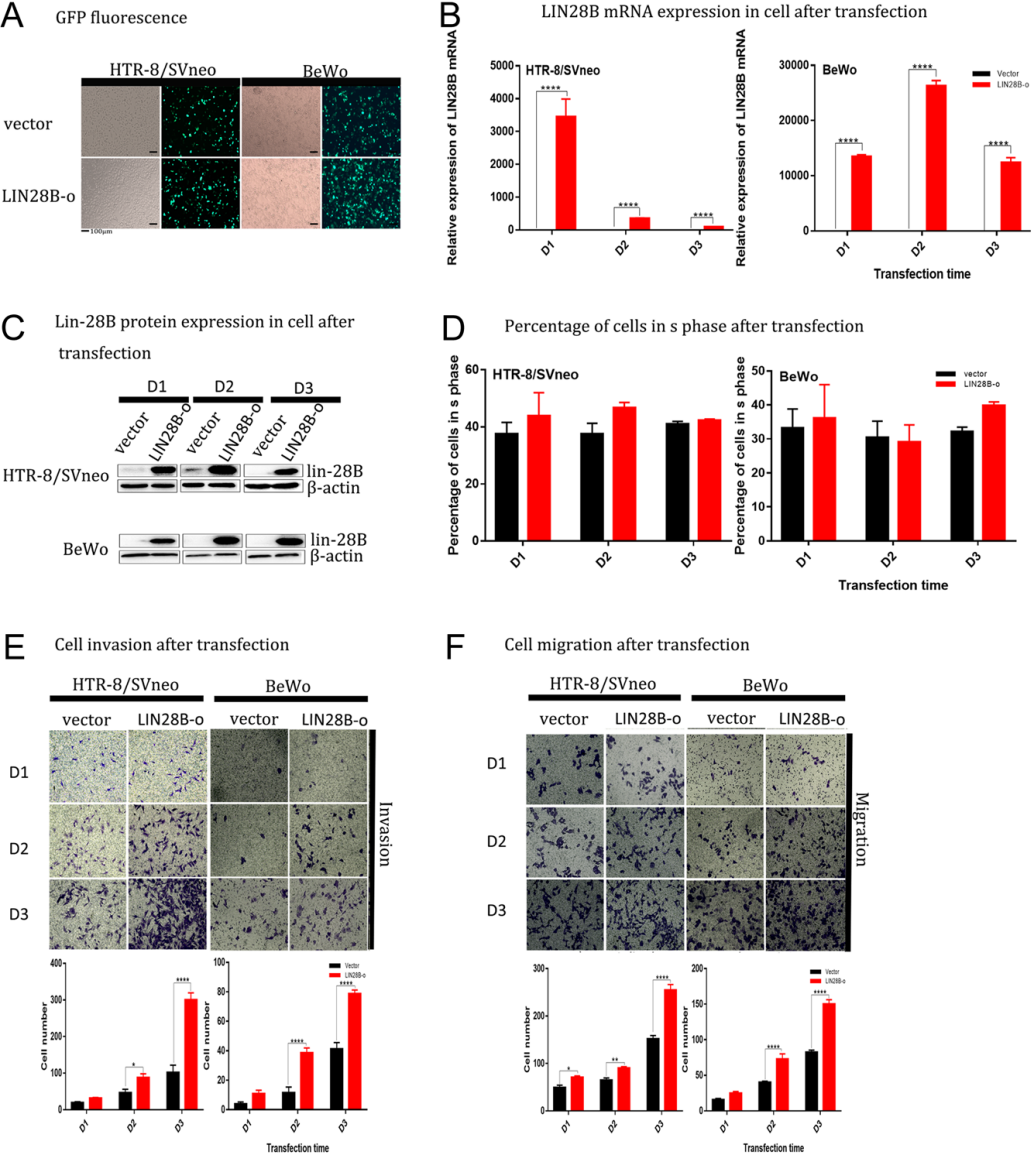
LIN28B promotes invasion and migration of HTR-8/SVneo and BeWo cells. (A) Transfection of HTR-8/SVneo and BeWo cells into empty vector control group (vector) and LIN28B overexpression plasmids (LIN28B-o) with fluorescence photos. (B and C) qPCR and Western blot analysis of vector and LIN28B-o groups after transfection of HTR-8/SVneo and BeWo cells into empty vector control group were performed on 1 day (D1), 2 days (D2), 3 days (D3). (D) The percentage of HTR-8/SVneo and BeWo cells transfected with vector and LIN28B-o plasmid D1, D2, D3 into S phase by flow cytometry. (E and F) The representative microphotographs of invasive cells in transwell invasion assay (plus Matrigel) and migration cells in transwell migration assay. The number of experimental replicates and the number of replicates within each experiment were both three times. ****P < 0.0001, ***P < 0.001, **P < 0.01, *P < 0.05.

To verify the possible role of LIN28B in placental villous trophoblastic cells from women with URSA, we selected two common cell lines that represent placental function (HTR-8/SVneo is primarily used to detect invasiveness and BeWo for fusion induction). Based on the low LIN28B expression of HTR-8/SVneo and BeWo, we constructed a LIN28B overexpression plasmid (LIN28B-o) and the corresponding empty vector (vector). One day (D1), 2 days (D2), and 3 days (D3) after transfection, we used fluorescence microscopy to quantify plasmid fluorescence (Fig. 2A), and the transfection effect was verified by qPCR (Fig. 2B) and Western immunoblotting analysis (Fig. 2C). Results showed that the expression of LIN28B in the overexpression group was significantly increased compared with the control group and that the cell model was successfully constructed.

### LIN28B promotes cellular migration and invasion

We found that invasion (Fig. 2E) and migration (Fig. 2F) of the LIN28B overexpression group increased in both HTR-8/SVneo and BeWo cells, but there was no significant difference in the proliferative capacity between the two groups (Fig. 2D). Cell cycle detection (Fig. 2D) showed that compared with the vector group, the proportion of cells entering S phase increased (HTR-8/SVneo, 37.52 ± 2.04 (D1), 37.5 ± 1.89 (D2), 40.97 ± 0.54 (D3); BeWo, 33.17 ± 2.13 (D1), 30.37 ± 1.58 (D2), 32. 16 ± 0.75 (D3) vs LIN28-o group: HTR-8/SVneo, 43.78 ± 4.13 (D1), 46.66 ± 0.94 (D2), 42.25 ± 0.31 (D3); BeWo, 36.15 ± 3.71 (D1), 28.71 ± 1.70 (D2), 39.8 ± 0.54 (D3)) (*P* > 0.05). No significant difference was observed in cell proliferation. The invasive ability of HTR-8/SVneo or BeWo cells in the LIN28B-o group was two to three times that of the control group (Fig. 2E), and the migratory capability was three times higher than that in the control group (*P* < 0.05) (Fig. 2E and F).

### LIN28B inhibits cellular apoptosis

**Figure 3:**
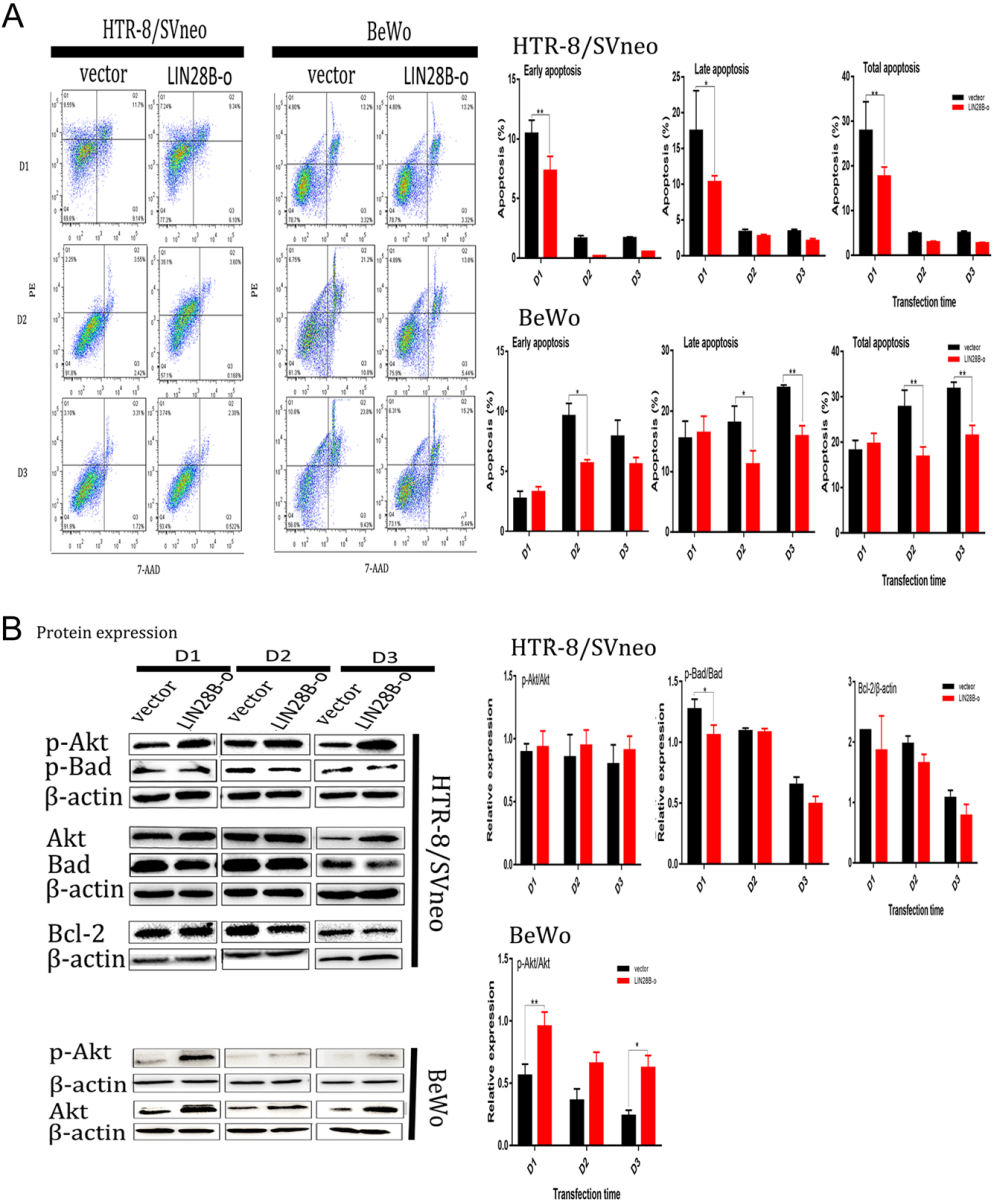
LIN28B inhibits apoptosis of HTR-8/SVneo and BeWo cells. (vector) and LIN28B overexpression (LIN28B-o) plasmids were transfected into empty vector control group after 1 day (D1), 2 days (D2), 3 days (D3). (A) Flow cytometry was used to determine the apoptosis of HTR-8/SVneo and BeWo cells by flow cytometry (FCM) staining with annexin V and 7-aminoactinomycin (7-AAD). The results showed that the percentage of apoptotic cells was early, late-stage, and total apoptotic cells (annexin V positive). (B) The expression of total protein and phosphorylated protein of Akt/Bad/Bcl-2 apoptosis-related pathway in HTR-8/SVneo and BeWo cells transfected with vector and LIN28B-o plasmids D1/D2/D3 was studied. The number of experimental replicates and the number of replicates within each experiment were both three times. ****P < 0.0001, ***P < 0.001, **P < 0.01, *P < 0.05.

Flow cytometry showed that the percentages of cells in early, late, and total apoptosis in the LIN28-o group were lower than in the vector group (Fig. 3A). In HTR-8/SVneo cells, only the difference on D1 was statistically significant (*P* = 0.002). In BeWo cells, the apoptotic index on D1 remained unchanged (*P* > 0.05), but the apoptosis on D2 (*P* = 0.03) and D3 (*P* = 0.003) significantly decreased. To further prove that LIN28B inhibits apoptosis, we confirmed protein expression for Akt/Bad/Bcl-2 and their phosphorylated states (Fig. 3B) and found that p-Akt in HTR-8/SVneo cells in the LIN28-o group increased (*P* > 0.05) and in p-Bad (*P* < 0.05) and Bcl-2 decreased (*P* > 0.05). In the LIN28-o group, only p-Akt increased in BeWo cells (*P* < 0.05).

### LIN28B inhibits cellular fusion

**Figure 4:**
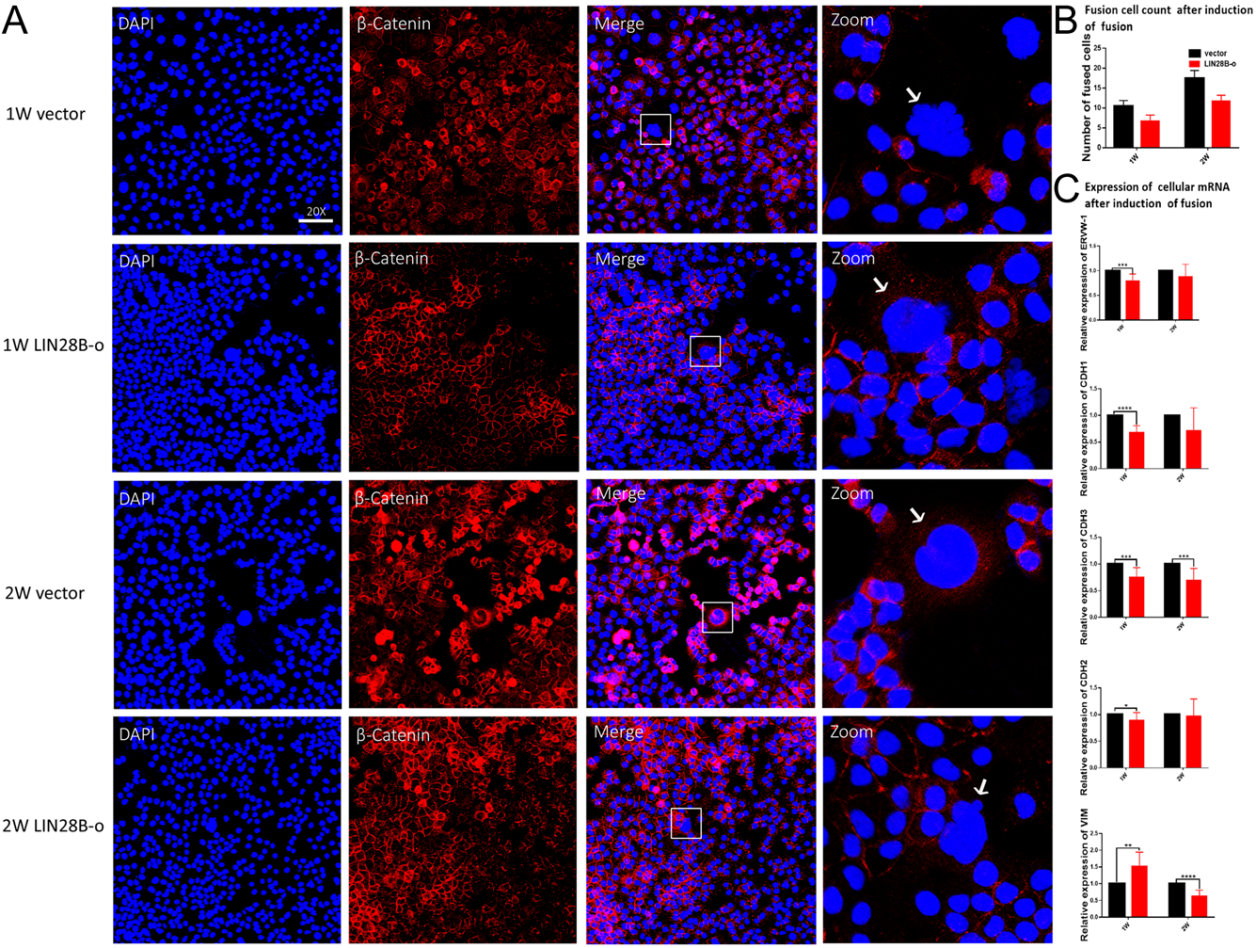
LIN28B inhibits cell fusion. BeWo cells were transfected with (vector) and LIN28B overexpression (LIN28B-o) plasmids in the blank vector control group. The fusion was induced by 50 μM FSK and 0.1% DMSO. After 1 or 2 weeks of continuous culture, immunofluorescence staining was carried out, and the corresponding RNA was observed or extracted by confocal microscope for qPCR test. (A) β-Catein labeled cell membrane (red, DAPI labeled nucleus (blue). (B) The number of fused cells (≥3 nuclear fusions) counted under confocal microscopy after induction of fusion. (C) qPCR was used to detect the mRNA expression levels of BeWo cell fusion-associated factors ERVW-1, CDH1, CDH3, CDH2, and VIM. The number of experimental replicates and the number of replicates within each experiment were both three times. ****P < 0.0001, ***P < 0.001, **P < 0.01, *P < 0.05.

Our study found that BeWo cells in the LIN28B-o group were more likely to inhibit cell fusion than cells in the vector group. After induction with 50 μM FSK, we observed with laser confocal microscopy that the number of cells fused at 1 or 2 weeks in the vector group was higher than that in the LIN28B-o group (Fig. 4A and B). To further verify that LIN28B was associated with cell fusion, we used qPCR to detect the expression of ERVW-1 (a factor known to be involved in cell fusion) and also evaluated E-cadherin (CDH1), P-cadherin (CDH3), vimentin (VIM), and N-cadherin (CDH2), factors related to epithelial-interstitial transformation. Our results showed that the expression of ERVW-1 in the vector group was higher than in the LIN28B-o group (*P* = 0.005) and that the expression of the epithelial-related factors CDH1 and CDH3 was lower than in the LIN28B-o group after induction with 50 μM FSK for 1 or 2 weeks (*P* < 0.05). VIM expression in the vector group after 1 week of induction was higher than that of the LIN28B-o group (*P* < 0.05), while the remaining indices showed a decreasing tendency (*P* < 0.05) (Fig. 4C).

## Discussion

In the present study, we have for the first time detected the expression of LIN28B in villi from women with URSA and have explored a possible role for LIN28B in URSA. LIN28B is a paternal imprinting gene (Davis *et al.* 2015) (i.e. expressed by the paternal source) that promotes the development of the placenta and also stimulates the embryo or fetus to extract nutrients from the mother, thereby promoting the growth of the individual (Moore 2001, Huppertz 2008, Monk 2015). Our study found that the expression of LIN28B in the villous tissue of URSA patients decreased, implying that the expression of the parental imprinting gene LIN28B may inhibit the development of trophoblast cells and lead to the occurrence of miscarriage.

Recent studies have shown that changes in trophoblastic function are realized through various signal transduction pathways (Chan *et al.* 2013, Seabrook *et al.* 2013, Lozoya *et al.* 2014, Lin *et al.* 2018, Canfield *et al.* 2019). LIN28 is highly expressed in the placenta and plays an important role in embryonic development and implantation as an RNA-binding protein (Canfield *et al.* 2019). Some studies have shown that the increase in LIN28B expression is positively correlated with the invasion, migration, and proliferation of trophoblast cells (Thornton & Gregory 2012, Shyh-Chang & Daley 2013, Park *et al.* 2017, West *et al.* 2019). Canfield *et al*. (2019) found that invasive interstitial EVT expressed higher levels of LIN28B in placental sections during early pregnancy compared with non-invasive proximal trophoblast cells, and an increased expression of LIN28B increased HTR8/SVneo cell proliferation, migration, and invasion* in vitro*. We demonstrated that HTR-8/SVneo and BeWo cells that overexpress LIN28B possessed enhanced migratory, and invasive capabilities, consistent with previous studies. Normal pregnancy requires adequate early EVT to invade the decidua, a situation highly similar to cancer metastasis with the proper remodeling of the spiral artery (Velicky *et al.* 2016, Chang *et al.* 2018, Pollheimer *et al.* 2018). Lin *et al.* (2018) found that LIN28B may inhibit apoptosis of ovarian cancer cells through the AKT2/FOXO3A/BIM axis. To further investigate the possible role of LIN28B in trophoblast cells, we also examined the apoptotic function and apoptosis-related proteins of HTR-8/SVneo and BeWo cells that overexpress LIN28B and discovered that anti-apoptotic ability was enhanced after overexpression of LIN28B, that the phosphorylation of the anti-apoptotic protein Akt increased, and that the phosphorylation of the pro-apoptotic proteins Bad and Bcl-2 decreased in HTR-8/SVneo cells. In BeWo cells, we only detected an increase in phosphorylation of the anti-apoptotic protein Akt, which may have been due to either extremely low or very unstable protein phosphorylation in BeWo cells after overexpression of LIN28B. We hypothesized that LIN28B inhibited apoptosis of villous cells through the Akt/Bad/Bcl-2 signaling pathway, thereby changing the migration and invasion of villi and ensuring the smooth progress of the pregnancy. The low expression of LIN28B in villous tissue of URSA patients may accelerate dysfunction and apoptosis in trophoblast cells, leading to the occurrence of abortion. But there were limitations of this experiment. Bad, Bcl2, and Akt not only affect the mechanism of apoptosis but also affect many other cell activities. More apoptosis evaluation experiments should be added, such as: adding apoptosis inhibitor (zVad-fmk), detecting apoptosis-related factors such as caspase-3 and PARP.

Cell fusion in mammals is a common physiologic process that is involved in fertilization, placental development, skeletal muscle and bone development, and immune defense responses (Pötgens *et al.* 2004, Deng *et al.* 2017). During the development of the human placenta, it undergoes primary synthesis and secondary integration. When cytotrophoblast cells are fused to the syncytiotrophoblast, they are regulated by various cytokines and growth factors (Maltepe *et al.* 2010, Gamage *et al.* 2016, Turco *et al.* 2018). Syncytin-1 (ERVW-1) was the first molecule to be ascribed a direct ability to promote cell fusion (Pötgens *et al.* 2004). Canfield *et al.* (2019) demonstrated that LIN28B plays a role in preeclampsia by reducing syncytialization and that JEG3-knockout of LIN28B in cells significantly decreased SYN-1 while LIN28B overexpression in HTR8/SVneo cells decreased TNF-α expression. Hypoxic culture significantly decreased the expression of LIN28B and SYN-1 in BeWo and EG3 cells and increased the expression of TNF-α. EMT is a biological process in which differentiated epithelial cells lose epithelial characteristics and acquire mesenchymal migration. This phenomenon not only plays an important role in tumor invasion and migration, formation of endoderm, and primitive intestinal lumen but also participates in cell-fusion processes (Asli & Harvey 2013, Pei *et al.* 2019). Lu *et al.* (2016) speculated that EMT may play a role in the trophoblast cell assembly process and that Twist1 promotes human placental tissue. Seabrook *et al.* (2013) showed that knockdown of LIN28A in human trophoblast-like ACH-3P cell lines induced spontaneous syncytialization in the early pregnancy. In the present study, we found that after overexpression of LIN28B the expression level of ERVW-1 decreased, the expression of E-cadherin and P-cadherin increased and that the expression of the interstitial-related factors vimentin and N-cadherin fluctuated greatly. The number of cells fused was also lower than in the control group, and cell-fusion ability was attenuated. Our results indicated that by overexpression of LIN28B and induction of fusion, cell epithelial characteristics increased, while mesenchymal cells fluctuated. This may be because EMT is a transient and reversible process, consistent with studies by Li *et al.* (2017) where the EMT expression at different time-points during hESC differentiation was at a dynamic level. Generally, secondary syncytium exerts obvious effects after 12 weeks of gestation, when the synthesized contracted trophoblast cells play an important role in providing nutrition and gas exchange. The invasive capability of EVT manifests a significant time limit that only occurs in the early pregnancy (Burrows *et al.* 1996, Chakraborty *et al.* 2002). Overexposure of cytotrophoblast cells in the early pregnancy, then, will result in the differentiation of invasive EVT cells, with a concomitant diminution in invasive ability accompanied by implantation failure and, ultimately, early abortion.

Several studies have shown that LIN28 plays a role in regulating stem cell activity, including self-renewal and differentiation. Therefore, during the early stages of embryonic development, early gene expression in the placenta is high, and cells continuously develop and differentiate (Park *et al.* 2017, West *et al.* 2019). LIN28 is also a key factor in the regulation of developmental differentiation. In the present study, we determined the expression of LIN28B in villous tissue from some patients with early URSA due to the inherent cellular capabilities for apoptosis, invasion, migration, and cell fusion. The cells *in vivo* exert their functions through various coordination mechanisms. This study has a certain limitation and cannot directly prove whether lin28b plays the same role* in vivo* as* in vitro* cells, but we will conduct further studies to prove the role of LIN28B* in vivo* cells or in URSA animal models. In the later stage, the peripheral blood expression of LIN28B-related miRNA Let-7 may be detected to predict or interfere with the occurrence of miscarriage. Our future aims are to further confirm that LIN28B is a contributing factor in early abortion diseases and participates in potential mechanisms underlying placental differentiation, development, and function, thus providing a foundation for further molecular research in this area.

## Conclusions

The expression of LIN28B is decreased with URSA, inhibiting cell invasion, and migration, and promoting apoptosis and fusion. Dystrophic dysfunction, then, may be one of the causes of miscarriage.

## Declaration of interest

The authors declare that there is no conflict of interest that could be perceived as prejudicing the impartiality of the research reported.

## Funding

This experiment was supported by funding from the National Nature Science Foundation of China (81300484); the Natural Science Foundation of Guangdong Province, China (2018A0303130308). The manuscript editing and publishing fees of this paper will be funded by the Guangdong provincial medical research fund (B2019007) and the Zhanjiang City Financial Fund Technology Competitive Fund (2019A01023).

## Ethics approval and consent to participate

The protocol governing the subjects of this study was approved by the Ethics Committee of the Affiliated Hospital of Guangdong Medical University (PJ2013014). All of the patients signed informed consent forms.

## Availability of data and materials

All data generated or analyzed during this study are included in this article.

## Author contribution statement

Q Y H: implementation of overall research, data analysis, and draft writing. Y R N and C X W: complete immunohistochemistry and cell culture. L J S and J Z H: collection of embryonic villi in aborted patients. T Z M: completed manuscript review, revised paper submission. All authors read and approved the final manuscript.
